# Vitamin D Supplementation for the Treatment of Acute Childhood Pneumonia: A Systematic Review

**DOI:** 10.1155/2013/459160

**Published:** 2013-12-19

**Authors:** Rashmi Ranjan Das, Meenu Singh, Inusha Panigrahi, Sushree Samiksha Naik

**Affiliations:** ^1^Department of Pediatrics, All India Institute of Medical Sciences (AIIMS), Bhubaneswar 751019, India; ^2^Department of Pediatrics, Post-Graduate Institute of Medical Education and Research (PGIMER), Chandigarh 160012, India; ^3^Department of Obstetrics & Gynecology, SCB Medical College, Cuttack 753007, India

## Abstract

*Background*. Studies have found an increased incidence of vitamin D deficiency in children with pneumonia; however, there is no conclusive data regarding the direct effect of vitamin D supplementation in acute pneumonia. *Methods.* A comprehensive search was performed of the major electronic databases till September 2013. Randomized controlled trials (RCTs) comparing treatment with vitamin D3 versus placebo in children ≤5 years old with pneumonia were included. *Results*. Out of 32 full text articles, 2 RCTs including 653 children were eligible for inclusion. One trial used a single 100,000 unit of oral vitamin D3 at the onset of pneumonia. There was no significant difference in the mean (±SD) number of days to recovery between the vitamin D3 and placebo arms (*P* = 0.17). Another trial used oral vitamin D3 (1000 IU for <1 year and 2000 IU for >1 year) for 5 days in children with severe pneumonia. Median duration of resolution of severe pneumonia was similar in the two groups (intervention, 72 hours; placebo, 64 hours). Duration of hospitalization and time to resolution of tachypnea, chest retractions, and inability to feed were also comparable between the two groups. *Conclusions*. Oral vitamin D supplementation does not help children under-five with acute pneumonia.

## 1. Introduction

Worldwide, acute lower respiratory tract infection (ALRTI) is a leading cause of mortality in children less than 5 years old [1, 2]. More than 90% are in developing countries. The management of ALRTI includes intravenous antibiotics, oxygen, or assisted ventilation (in severe cases). Besides these, nutritional supplementations such as zinc and vitamin A supplementation have been tried, though the results have been unfavorable [3, 4]. Researchers have found that deficiency in vitamin D may predispose people to infection, and thus vitamin D has been labeled as antibiotic vitamin [5]. The immune enhancing actions of vitamin D include induction of monocyte differentiation, inhibition of lymphocyte proliferation, stimulation of phagocytosis dependent and antibody-dependent macrophages, and modulation of T and B lymphocytes that produce cytokines and antibodies [5–8]. Vitamin D deficiency if severe leads to chest wall deformity, hypotonia, poor chest wall compliance, atelectasis, and fibrosis [9]. All these factors contribute to a higher incidence of pneumonia in children with severe vitamin D deficiency.

A recent meta-analysis of randomized controlled trials (RCTs) showed that *prophylactic* vitamin D supplementation in the pediatric age group reduced the rate of respiratory tract infections significantly (odds ratio (OR), 0.58; 95% CI, 0.41–0.8) [10]. Few studies have assessed the *therapeutic* efficacy of vitamin D supplementation as an adjunctive to antibiotics and supportive measures in treating childhood pneumonia. Unlike the evidence favoring a prophylactic effect, there is no clear evidence to support or refute the therapeutic efficacy of vitamin D in acute pneumonia. Hence the current meta-analysis was planned to detect whether vitamin D supplementation has any role in treatment of children <5 years old with acute pneumonia.

## 2. Methods

### 2.1. Criteria for considering Studies for This Review

#### 2.1.1. Types of Studies

Studies are randomized double-blind placebo-controlled trials (RCTs).

#### 2.1.2. Types of Participants

Participants are children of both sexes and >1 month to ≤5 years old, hospitalized with clinical diagnosis of acute pneumonia. Pneumonia was defined as age-specific tachypnea (>60/min if <2 months; >50/min if 2–11 months; >40 if 12–24 months) along with crepitations and absence of wheeze (with or without fever). Severe pneumonia was defined as age-specific tachypnea along with chest retractions or any of the danger signs (cyanosis, unable to feed, and lethargy). Studies including children suffering from other debilitating diseases, with severe wasting (weight for height <3SD), and known asthmatics were excluded.

#### 2.1.3. Types of Interventions

The intervention commenced after the child is hospitalized, and it consisted of treatment with vitamin D or placebo as an adjuvant to standard hospital treatment (including antibiotics, oxygen, and other supportive measures). The trials had to compare vitamin D with placebo only. All formulations of vitamin D were considered.

#### 2.1.4. Types of Outcome Measures

Outcome measures frequently used to determine the clinical efficacy of any acute pneumonia treatment are the duration of resolution illness, duration of hospitalization, treatment failure, adverse events, or death. Accordingly, trials measuring following outcomes were included in the review. 

*Primary Outcome Measures*

1. Time to resolution of severe illness.
2. Duration of hospitalization.

*Secondary Outcome Measures*

1. Time to resolution of
  - tachypnea,
  - chest retractions,
  - hypoxia (SpO_2_ < 95%),
  - fever,
  - inability to feed/lethargy.
2. Adverse-events.

Time to resolution of acute pneumonia was defined as the time period (hours/day) to achieve the following parameters from initiation of treatment: respiratory rate less than the age-specific cut-offs, no chest indrawing, no danger signs or hypoxia, and ability to feed, for two consecutive days. The duration of hospitalization was defined as the time (hours/days) between study enrollment and discharge. Treatment failure was defined as no reduction in the tachypnea over 72 hrs period compared to that detected at enrolment.

### 2.2. Search Methods for Identification of Studies

We searched the Cochrane Central Register of Controlled Trials (CENTRAL) (*The Cochrane Library*, Issue 2), which contains the Cochrane Acute Respiratory Infection (ARI) Group and the Cochrane Infectious Diseases Group Specialized Registers; MEDLINE (1970 to September week 4, 2013); EMBASE (1974 to July 2013). For MEDLINE search, following search terms were adopted: ((*exp Pneumonia/*)* OR pneumonia OR lower respiratory tract infection$ OR LRTI OR lower respiratory infection$*)* AND *(*exp Vitamin D/OR vitamin D OR Cholecalciferol OR cholecalciferol OR Ergocalciferol OR ergocalciferol*)* AND *(*exp Vitamin/OR vitamin*)* AND *(exp Child/OR child OR children exp Infant/OR infant OR infants OR paediatric OR pediatric OR Preschool/OR toddler OR preschool*). To identify RCTs, whose results had remained unpublished, we searched the NIH clinical trial register (http://www.clinicaltrials.gov/) and found no trial regarding the use of vitamin D in treatment of acute pneumonia (access on 5th December 2013). Two independent reviewers (RRD, IP) reviewed the search results to identify relevant original human clinical or field trials. Studies that focused on the prophylactic effects of vitamin D in acute respiratory infections were excluded from the analysis. Additional studies were identified through manual searches of reference lists of the originally identified studies on the therapeutic and preventive roles of vitamin D, as well as reviews on the subject. No language restrictions were applied.

### 2.3. Data Collection and Analysis

#### 2.3.1. Assessment of Risk of Bias in Included Studies

Two review authors (RRD, SSN) independently assessed the methodological quality of the selected trials by using methodological quality assessment forms. We undertook quality assessment of the trials using the criteria outlined in the *Cochrane Handbook for Systematic Reviews of Interventions* [11]. Any disagreements between the two review authors were resolved by discussion with the third author (MS). Trials were assessed with respect to the extent to which investigators minimised the potential for bias to occur and addressed other issues in relation to methodological quality. When the methodological description was unambiguous, one review author entered the methodological description to the “Risk of bias” tables in characteristics of included studies. When the description of methods was ambiguous, the same review author discussed the issue with the coauthor to reach a consensus. The potential for selection (systematic differences in the comparison groups), performance (systematic difference in the care provided apart from the intervention being evaluated), exclusion (systematic differences in withdrawals from the trial), and detection (systematic differences in outcome assessment) bias was assessed.

#### 2.3.2. Study Descriptions

Information in relation to methodological quality, characteristics of participants, interventions, and outcome measures of each trial is provided in Table 1.

#### 2.3.3. Assessment of Heterogeneity

It is not applicable, as the result could not be pooled.

#### 2.3.4. Data Synthesis

Continuous data were expressed as mean ± standard deviation (SD) and standardized mean difference (SMD) was obtained. Dichotomous data were expressed as odds ratio (OR) with 95% CI. *P*-value < 0.05 was considered significant.

## 3. Results

### 3.1. Description of Studies

**Figure 1:**
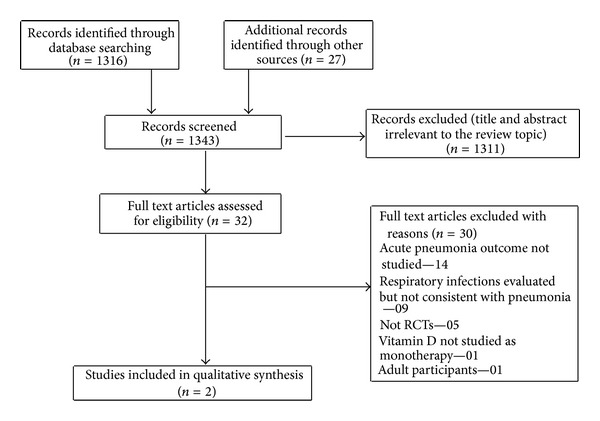
Flow diagram of search results. RCTs: Randomized controlled trials.

Of 1343 citations retrieved, full texts of 32 articles were assessed for eligibility (Figure 1). Out of these, a total of 30 articles were excluded for the following reasons: acute pneumonia outcomes not studied (*n* = 14), respiratory infections evaluated but not consistent with pneumonia (*n* = 09), not RCTs (*n* = 05), not studied vitamin D as monotherapy (*n* = 01), and included adult participants (*n* = 01). Finally, 2 studies (including 653 children) were considered as potentially eligible for inclusion (Table 1) [12, 13]. Both the included studies were conducted in developing countries (India = 1, Afghanistan = 1). As vitamin D deficiency is common in both the countries, the characteristics of study population, as well as etiological profile of pneumonia, are supposed to be more uniform in the included studies. But, the two studies were heterogeneous regarding the vitamin D dosage, the duration of the treatment, the age of children enrolled, and also the severity of pneumonia. Both the trials used WHO and IMCI (integrated management of childhood illnesses) protocol for classification as well as case management of pneumonia. Children >1 month (not neonates) were included in the studies. The dose of vitamin D used in these trials varied from a total dose of 5,000–100,000 IU with duration of use being 1–5 days. One trial included children with both severe and nonsevere pneumonia [12], whereas the other included only severe pneumonia cases [13].

### 3.2. Risk of Bias in Included Studies

This has been described in Table 2. From this risk of bias tool it can be predicted that both the trials are of good qualities having low risk of bias.

### 3.3. Effect of Interventions

#### 3.3.1. Primary Outcome Measures

*(1) Duration for Resolution of Pneumonia*. Both the trials reported this outcome. In one trial, there was no significant difference in the mean (±SD) number of days to recovery from pneumonia between the vitamin D3 (4.74 ± 2.22) and placebo arms (4.98 ± 2.89) (*P* = 0.17) [12]. In the other trial, the median duration (SE, 95% CI) of resolution of severe pneumonia was similar in the two groups (intervention: 72 (3.7, 64.7–79.3) hours; placebo: 64 (4.5, 55.2–72.8) hours) (*P* = 0.33) [13].

*(2) Duration of Hospitalization*. One trial reported this outcome [13]. The median duration (IQR) of resolution of severe pneumonia was similar in the two groups (intervention: 112 (96–136) hours; placebo: 104 (88–128) hours) (*P* = 0.29).

#### 3.3.2. Secondary Outcome Measures

Only one study including children with severe pneumonia reported all the following outcomes [13].

*(1) Duration of Resolution of*

- Tachypnea: the median (IQR) duration of resolution was similar in the two groups (intervention: 72 (56–104) hours; placebo: 72 (48–98) hours) (*P* = 0.33).
- Chest retractions: the median (IQR) duration of resolution was similar in the two groups (intervention: 64 (40–88) hours; placebo: 64 (40–88) hours) (*P* = 0.38).
- Hypoxia (SpO_2_ < 95% on room air): the median (IQR) duration of resolution was similar in the two groups, (intervention: 16 (8–24) hours; placebo: 16 (8–24) hours) (*P* = 0.86).
- Fever: the median (IQR) duration of resolution was similar in the two groups, (intervention: 80 (64–104) hours; placebo: 72 (56–104) hours) (*P* = 0.52).
- Inability to feed/lethargy: the median (IQR) duration of resolution was similar in the two groups (intervention: 64 (48–88) hours; placebo: 56 (48–72) hours) (*P* = 0.21).

*(2) Adverse-Events. *No major adverse-events were reported in both the trials. In one trial, 2 minor adverse events were reported that included one episode of vomiting and diarrhea lasting for 2 days in the intervention group only [13].

## 4. Discussion

### 4.1. Summary of Evidence

After an extensive search of the literature we could find only 2 trials to be eligible for inclusion in the present systematic review. Our result indicates that vitamin D3 supplementation has no effect on the time period to resolution or recovery from pneumonia, duration of hospitalization, time to resolution of tachypnea, chest retractions, and inability to feed/lethargy. We could not carry out meta-analysis because of lesser number of trials.

Both the trials did not show a beneficial effect of vitamin D3 supplementation in acute (severe and nonsevere) pneumonia, though both were adequately powered. Then, what might be the reason for ineffectiveness of vitamin D3 supplementation in treatment of acute pneumonia. Is it because of the improper dose or the duration, or the real therapeutic ineffectiveness of vitamin D in acute pneumonia? All these are important points and need to be addressed now, so that the future trials can be given some direction. Otherwise, we have to wait for a longer period to find out the actual evidence. First, regarding the dose schedule, it varied between the two trials. One trial used a single bolus dose of 100,000 IU [12], and the other [13] used doses varying from 5,000 to 10,000 IU. It has been shown that a daily dose schedule has a better therapeutic effect than a large bolus dose [14], and there have been biological explanations to a smaller effect when using a bolus schedule of vitamin D [15–17]. In fact, vitamin D is immunosuppressive at higher doses. A trial that used 10,000 IU/day of vitamin D clearly showed suppressed proliferative responses of peripheral blood monocytes [18]. In one of the included trials studying simultaneously the prophylactic effect of vitamin D using large bolus doses (100,000 IU every 3 months), the intervention group had a slightly higher risk of secondary pneumonia [12]. However, the other included trial using a daily dose schedule also did not find a beneficial effect in severe pneumonia [13]. But in this trial, an inadequately low dose might have been used considering the wide prevalence of underlying vitamin D deficiency in the studied population [19]. Ideally, measurement of vitamin D level would have been meaningful in relation to the interpretation of result, but neither of the included trials measured the level because of financial constraints. The No Observed Adverse Effect Level (NOAEL; 2400 IU) and Lowest Observed Adverse Effect Level (LOAEL; 3800 IU) of vitamin D have been previously defined [20]. So, future trials should use a different dose schedule keeping these in mind, and it would be better to measure the blood vitamin D level at baseline and at completion of the trial to corroborate with the outcomes as well as with toxicity (if any). Second, regarding the real therapeutic ineffectiveness of vitamin D in acute pneumonia, it would be too early to make any comment. The answer might come from ongoing or future community and hospital based trials.

The prevalence of malnutrition was not reported in any of the included trials, though they excluded children with severe malnutrition. It has been hypothesized that malnutrition might affect the state of immunity and hence the effect of vitamin D. The children from developing countries commonly have lower blood vitamin D levels. So the explanation can be both the ways: preexisting vitamin D deficiency, making the child susceptible to pneumonia due to impaired immunity in one hand, and pneumonia causing lower vitamin D levels due to acute inflammatory response on the other hand. Previously, vitamin D level has been shown to decrease during acute inflammation in human studies [21]. Monitoring of vitamin D level might help to understand the actual response that would guide us about the optimal/therapeutic dose to be used in acute pneumonia.

The etiology of pneumonia in the included studies might also have varied with age, region, and time/season of study. But microbiological studies were not performed in any of the included trials. It has been found that viral pneumonias dominate over bacterial ones in the rainy season. At least one-year study period would therefore be meaningful as per the etiological agents are concerned. However, the study period was only 2 months in one trial [12] and not even mentioned in the other trial [13]. So, it is difficult to interpret the ineffectiveness of vitamin D in pneumonia caused by any particular agent (bacterial or viral). The outcome results might also have varied depending on the timing of institution of therapy with vitamin D in the course of pneumonia. So, the duration of illness prior to administration of vitamin D would be more meaningful and should be included in future trials. Depending on the study setting, different proportions of enrolled children might have received antibiotics prior to enrollment into the trial. Prior antibiotic exposure can modify recovery, and information on prior antibiotic use must be included in the baseline data in order to allow for comparisons between trials, which were not given in any of the included trials. Similarly, large proportion of study subjects needing “second-line” or “broader spectrum” antibiotics due to severe illness would lead to a reduction in power, and it may be difficult to detect any differences caused by vitamin D in the two groups. Field trials or trials at primary or secondary healthcare level on less severe cases of pneumonia with less exposure to higher antibiotics may be more useful in demonstrating the effect of vitamin D.

### 4.2. Limitations

Only two RCTs were included to generate the evidence. We could not take into account the etiologies (bacterial or viral or both) of pneumonia to report beneficial effect of vitamin D (if any), as they were not reported in the included trials. As vitamin D level was not measured in any of the trials, it was difficult for us to make any suggestion about the dose-response effect in pneumonia. Our results may not be applicable to children with HIV infection, severe malnutrition, neonates, and children in developed country.

### 4.3. Further Area of Research

Future trials should report about the etiological/microbiological diagnosis of acute pneumonia. The dose of vitamin D varied among both the trials. So, the future trials should focus on studying a higher dose preferably given in a daily schedule and for varying duration of pneumonia. Simultaneously, they should measure the vitamin D level to corroborate the clinical findings. Besides these, data on prior antibiotic use and duration of illness prior to vitamin D supplementation should also be provided. Finally, the trials (if possible) should also include a subgroup of children with severe malnutrition and/or rickets and those with wheezing.

## 5. Conclusion

To conclude, present data do not support therapeutic vitamin D supplementation in the management of under-five children (excluding the neonates) with acute pneumonia. Future trials should focus on the limitations/weakness identified in the present systematic review so that good quality evidence can be generated.

## Figures and Tables

**Table 1:** Characteristics of included studies.

Study	Setting	Participants	Intervention	Outcomes measured	Comments
Manaseki-Holland et al. [12]	Outpatient of inner-city hospital, Afghanistan.	[Vitamin D = 224, placebo = 229]. Age 1–36 months with acute pneumonia.	A single 100,000 unit of oral vitamin D3 at onset of pneumonia.	Time to resolution of pneumonia/recovery for 48 consecutive hours, treatment failure, and discharge from hospital.	Children with wheezing were excluded. No adverse effects noted. Microbiological and/or radiological diagnosis not done. Serum vitamin D3 level was not measured.
Choudhary and Gupta [13]	Inpatient of a tertiary care hospital, India.	[Vitamin D = 100, placebo = 100]. Age 2 months–5 years hospitalized with severe pneumonia.	Oral vitamin D3 (1000 IU for <1 year and 2000 IU for >1 year) for 5 days.	Primary: time to resolution of severe pneumonia (absence of lower chest indrawing, hypoxia or cyanosis, lethargy, and inability to feed). Secondary: duration of hospitalization and time to resolution of tachypnea, chest retractions, and inability to feed.	Sixty-three children had past history of pneumonia. Five children had clinical evidence of rickets. One-third of children had wheezing at enrollment. No major adverse effects noted. Microbiological and/or radiological diagnosis not done. Serum vitamin D3 level was not measured.

**Table 2:** Assessment of risk of bias by using Cochrane risk of bias tool.

Risk of bias parameters	Manaseki-Holland et al. [12]	Choudhary and Gupta [13]
Adequate sequence generation	Yes	Yes
Allocation concealment	Yes	Yes
*Blinding *	Yes	Yes
*Incomplete outcome data *	Yes	Yes
*Selective reporting *	No	No
*Other potential sources of bias *	No	No
